# Exact simulation of classical heat engine cycles using single-ion phonon laser

**DOI:** 10.1016/j.fmre.2024.01.008

**Published:** 2024-03-05

**Authors:** Q. Yuan, J.-Q. Zhang, Y.-Q. Wei, S.-Q. Dai, P.-D. Li, J. Li, T.-H. Cui, F. Zhou, L. Chen, J. Lin, M. Feng

**Affiliations:** a State Key Laboratory of Magnetic Resonance and Atomic and Molecular Physics, Wuhan Institute of Physics and Mathematics, Innovation Academy of Precision Measurement Science and Technology, Chinese Academy of Sciences, Wuhan 430071, China; b School of Physics, University of the Chinese Academy of Sciences, Beijing 100049, China; c Laboratory of Quantum Science and Engineering, South China University of Technology, Guangzhou 510641, China; d Research Center for Quantum Precision Measurement, Guangzhou Institute of Industrial Technology, Guangzhou 511458, China; e Department of Physics, Zhejiang Normal University, Jinhua 321004, China

**Keywords:** Heat engine, Trapped ions, Phonon laser, Carnot cycles, Otto cycles, Nonlinear vibrational mode, Thermodynamics, Duffing oscillator

## Abstract

Heat engines are essential devices in modern industry, converting heat energy into useful mechanical work via their working substances. Here we experimentally simulate the conventional heat engine by employing the vibrational mode of a single trapped ion as the working substance. In contrast to simply employing the ion in thermal motion, we consider coherently stimulating the ion’s vibrational motion as the phonon laser, which helps acquire clearer results by effectively suppressing the thermal fluctuation. As such, we demonstrate in an exact and high signal-to-noise way the standard steps of both the Otto and Carnot cycles in a single ion, and compare their maximum efficiencies by monitoring the amplitude and frequency of the vibration. Our work witnesses an interesting single-atom thermal engine using coherently controlled phonons. It would be the smallest platform for simulating or demonstrating classical thermodynamic laws and phenomena at a single ion scale via optical manipulation techniques for phonon lasers.

## Graphical abstract

**Figure:**
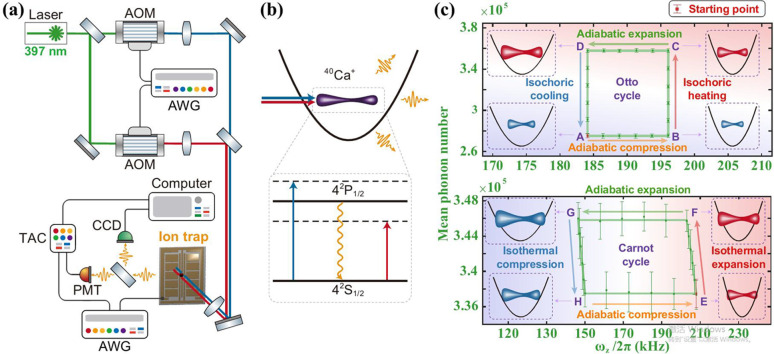

## 1 Introduction

Traditional heat engines are macroscopic devices with working substances containing a large number of particles, while advances in microtechnology have made it possible to reduce the sizes of heat engines to the microscale, even to the nanoscale [1], [2], [3], [4], [5], [6], [7], [8]. Recent studies have revealed that the heat-work conversion in nanoscale heat engines would deviate from the thermodynamic limit due to thermal and quantum fluctuations as well as non-equilibrium dynamics involved [8], [9], [10], [11], [12], [13], implying the failure of the assumptions of classical thermodynamics at the microscale. These counterintuitive results lead to extensive attention to the exploration of efficient and powerful microscopic heat engines with quantum properties. Until now, various physical systems, ranging from solid-state to atomic systems, have demonstrated the implementation of quantum heat engines [12], [13], [14], [15], [16], [17], [18], [19], [20], [21], [22], [23], [24], [25], [26], [27], [28], [29], [30], [31]. In contrast, we have also noticed some investigations of classical heat engines using, such as piezoresistive systems [1], colloidal particles [2], single-ion systems [4], Brownian particles [6], and cantilevers [32]. Although these heat engines present comparable-scale thermal fluctuations and follow stochastic thermodynamics, they still follow the predictions of classical thermodynamics due to their heating and cooling relevant to the classical thermal baths and their dynamics governed by the classical thermodynamics. However, due to thermal fluctuations and quantum fluctuation dominant in the dynamics, none of the above systems follows exactly the classical thermodynamics, and the experimental heat cycles largely deviate from the theoretical ones.

In this work, we report an experimental simulation of classical heat engine cycles using a cold trapped ion behaving as the phonon laser [33], [34], [35], [36], [37], [38], [39], [40]. Phonon lasers are mechanical analogs of optical lasers, which are employed to achieve coherent amplification of phonons with ubiquitous characteristics of optical lasers, such as the necessary threshold, the narrow linewidth, and nonlinear saturation effects [33]. Recent advances in phonon lasers are fueled by their potential in ultra-sensitive measurements and sensors [41], [42], [43], [44], [45]. These works are triggered by the pioneering experiments of detecting ultraweak oscillating forces [46]. However, more applications of the phonon laser still remain largely unexplored.

In our work, we extend the trapped-ion phonon laser to another application by employing the vibrational mode of the ion as the working substance of the heat engine. The key point of the heat engine is to coherently stimulate the vibrational mode to be the state of an injection-locked phonon laser [41], [42], [43], [44], [45], [46], i.e., an amplitude-amplified harmonic oscillation with the frequency locked. Instead of just employing the vibrational mode in a thermal state like in [8], we utilize the phonon laser (i.e., with $10^{5}$ phonons in coherent vibration) to acquire results with high signal-to-noise since the thermal fluctuations are effectively suppressed. As elucidated later, this phononic heat engine, although executed in a single ion, could exactly simulate standard processes of classical Carnot and Otto cycles and demonstrate the conventional thermodynamic phenomena, such as, the Carnot cycle takes a higher efficiency than the Otto one when their heat baths share the same temperatures. In our experiment, the two laser fields employed to produce the phonon laser, together with the real environment, constitute the baths, where the hot bath refers to the blue-detuned irradiation being dominant and the cold bath corresponds to the dominant red-detuned irradiation plus the environment (clarified later). Thermodynamic quantities are quantized based on the frequency and amplitude of the phonon laser. Our results indicate the feasibility of exploring classical thermodynamical properties at the single-atom level via engineering the vibrational degrees of freedom.

## 2 Single-ion phonon laser heat engine

**Fig. 1:**
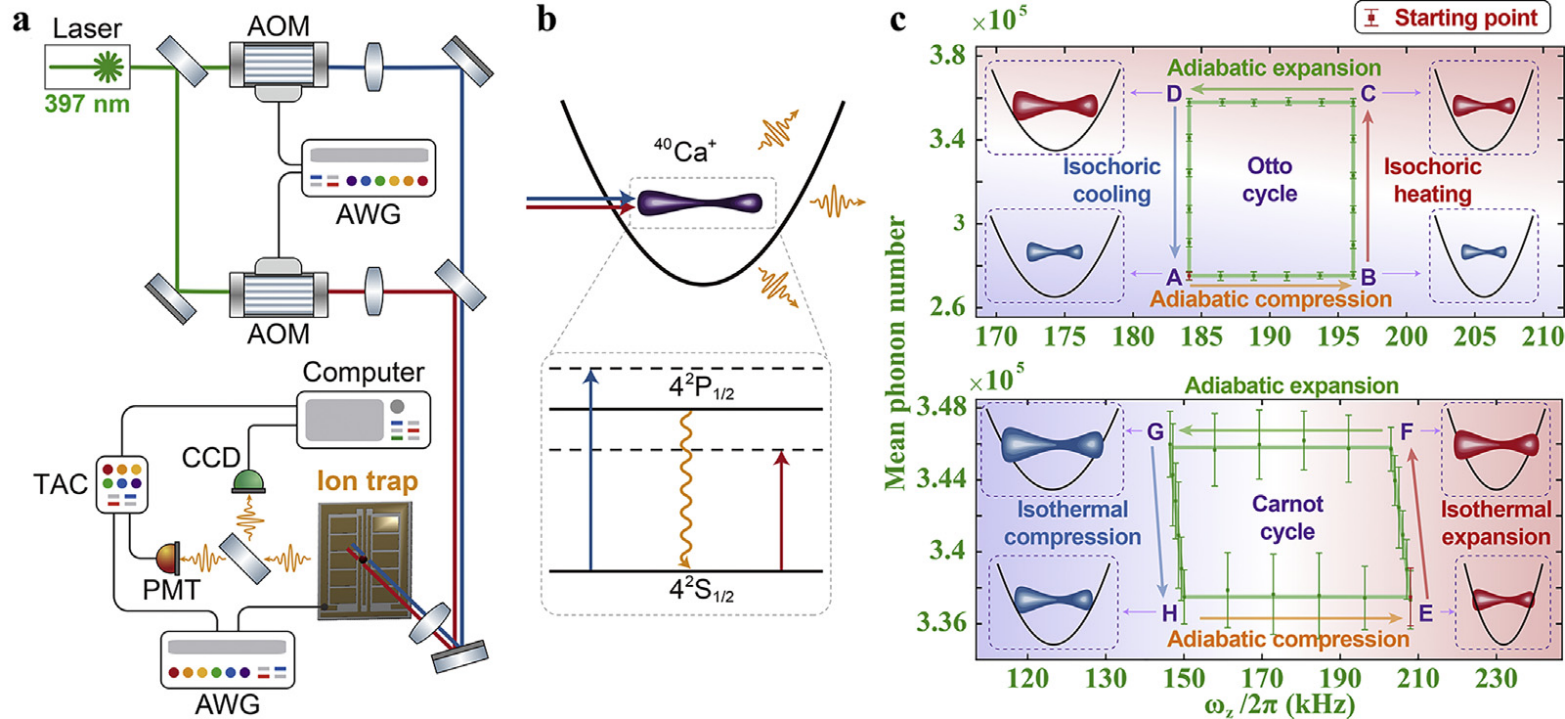
**Experimental setup and results for classical hear engine cycles.** (a) Schematic of the experimental setup. AOM: acousto-optic modulator. PMT: photomultiplier tube. AWG: arbitrary waveform generator. CCD: charge-coupled device. TAC: time to amplitude convertor. (b) Sketch of the phonon laser along with the level scheme of ${}^{40}$Ca${}^{+}$ ion under irradiation of the lasers, where the solid arrows represent the transitions by lasers with 397 nm wavelengths in blue and red detunings, and the wavy arrow represents the spontaneous emission. (c) Upper panel is for Otto engine cycle, with the strokes from A to B and from C to D corresponding to adiabatic processes, and the strokes from B to C and from D to A representing isochoric processes. Bottom panel is for Carnot engine cycle, with the strokes from E to F and from G to H corresponding to isothermal processes and the strokes from F to G and from H to E indicating adiabatic processes. The green solid lines represent the ideal engine cycles, and the dots mean the experimental data. The two cycles get started from A and E, respectively. The error bars indicate the statistical standard deviation of the experimental data from 40 measurements with each measurement time of 15 s.

Our experiment is carried out by a single trapped ${}^{40}$Ca${}^{+}$ ion in a surface-electrode trap (SET), as sketched in Fig. 1a, which is a 500-μm-scale planar trap as introduced previously [47], [48], [49]. The ion is first Doppler-cooled, staying at 800 μm above the surface of the SET, and then excited to be the state of a phonon laser in the potential well. The phonon laser refers to the oscillation amplification of the ion stimulated by two 397 nm laser beams, one of which is a red-detuned beam with detuning $\Delta_{r}/2\pi$ = -80 MHz and the other of which is a blue-detuned beam with $\Delta_{b}/2\pi$ = 40 MHz. Both of the laser beams are elaborately tuned to be with the appropriate intensity ratio r = $I_{b}/I_{r}$, where the oscillation amplification is proportional to r, see Fig. 1b and Fig. S1 in Ref. [52]. Besides, the phonon laser is solely related to the z−axis motional degree of freedom of the ion due to the frequency of $\omega_{z}/2\pi =183.5$ kHz being much smaller than the ones as $\omega_{x}/2\pi =532.1$ kHz and $\omega_{y}/2\pi =838.5$ kHz in other directions. The frequency differences of $(\omega_{x}-\omega_{z})/2\pi =348.6$ kHz and $(\omega_{y}-\omega_{z})/2\pi =655$ kHz ensure the motion in the z−axis decoupling from the ones in the x−axis and y−axis. Throughout the work, the phonon laser’s oscillation frequency is locked by applying an appropriate injection locking signal to the SET, which also has no influence on the other two directions. The dynamics of such a system is given by a Van der Pol equation:

(1) $$m\frac{d^{2}}{dt^{2}}z+m\gamma_{\mathrm{eff}}\left(z\right)\frac{d}{dt}z+m\omega_{z}^{2}z=F\sin\left(\omega_{l}t\right)$$

where m is the mass of the ion, $\gamma_{\mathrm{eff}}\left(z\right)$ is the effective loss/gain rate relevant to the red- and blue-detuned 397 nm lasers as well as the position z of the ion, and F and $\omega_{l}$ are the strength and frequency of the applied injection locking signal. The trap frequency $\omega_{z}$ is also the frequency of the phonon laser. Throughout the work, we set $\omega_{l}=\omega_{z}$, i.e., $\omega_{l}$ is varied when we change $\omega_{z}$. Note that the thermal fluctuations are omitted in the above equation since they are negligible compared to the strength of the phonon laser [33]. The working substance of the heat engine can be expressed as $H=m\omega_{z}^{2}A^{2}$, with A being the amplitude of the z-axis oscillation, i.e., the amplitude of the phonon laser.

Although the phonon laser behaves as coherent oscillation rather than thermal motion, we may employ it to simulate the Otto and Carnot cycles of the conventional heat engine by defining the characteristic quantities as [8], [50], [51]: The mean phonon number $\langle n\rangle =m\omega_{z}A^{2}/\hbar$, the temperature $T=m\omega_{z}^{2}A^{2}/k_{B}$, the entropy $S=\frac{k_{B}}{2}\left[1+\ln\left(\frac{k_{B}T}{\hbar \omega_{z}}\right)\right]$, the volume $V=\omega_{z}^{-1}$, and the pressure $P=m\omega_{z}^{3}A^{2}$, here $k_{B}$ denotes the Boltzmann constant [52]. In the adiabatic strokes, both the mean phonon number n and entropy S remain constant, but $\omega_{z}$ would change, leading to the variations of other quantities, e.g., P, T, and V, and thus performing non-zero work. In the isochoric strokes of the Otto cycle, V and $\omega_{z}$ remain unchanged, while the heat exchange between the working substance and the heat baths varies the quantities P, T, 〈n〉, and S. As such, no work is done in the isochoric strokes, and the working substance finally evolves to thermal equilibrium with the heat bath. In the isothermal strokes of the Carnot cycle, the temperature T should remain constant, and the working substance is always in thermal equilibrium with the isothermal heat bath. But the shift of $\omega_{z}$ generates the non-zero work due to the variations of P, and V, along with the change of 〈n〉 and S.

We can acquire the above defined quantities by experimentally engineering $\omega_{z}$ and measuring A. In our experiment, after inputting the injection-locking signal, we vary $\omega_{z}$ by tuning the electrode voltage and evaluate $\omega_{z}$ by observing the resonance signal (for $\omega_{z}=\omega_{l}$). The values of A are acquired by fitting the recorded photons scattered from the ion [45], where the scattering rate due to irradiation of the 397-nm laser beams is calculated along with a Gaussian term concerning the experimental noises by a convolution function [52].

## 3 Results and discussion

*Otto cycles*. We have performed two sets of experiments following the conventional procedure of the Otto and Carnot cycles, respectively, as sketched in Fig. 1c, and then compare the work, heat, and efficiency between the two sets of engine cycles. We first simulate the Otto cycle with the four strokes executed as follows: The isochoric strokes are accomplished by modifying the effective loss/gain rate $\gamma_{\mathrm{eff}}$ via tuning the strength of the blue-detuned 397 nm laser. The dominant blue- (red-)detuned irradiation results in smaller (larger) values of $\gamma_{\mathrm{eff}}$ and larger (smaller) values of A, reflecting the fact that the heat engine absorbs (releases) heat from (to) the hot (cold) bath. The adiabatic strokes are performed by tuning $\omega_{z}$, which generates non-zero work. Similar to previously proposed heat engines [6], [8], where the working substance always couples to the baths, we may replace the adiabatic strokes (i.e., with the mean phonon number 〈n〉 to be constant) with the isentropic strokes (i.e., with the ratio $T/\omega_{z}\propto \omega_{z}A^{2}\propto \langle n\rangle$ remaining constant) in our experimental operations, ensuring fixed entropies.

Our experiment starts from a steady state at A ($\omega_{\min}^{O},n_{\min}^{O}$), where our working substance takes the minimal frequency $\omega_{z}^{O}=\omega_{\min}^{O}$, the minimal phonon number $\langle n\rangle =n_{\min}^{O}$, but with the maximal amplitude $A_{\max}^{O}$. Then, we perform the adiabatic compression stroke from A ($\omega_{\min}^{O},n_{\min}^{O}$) to B ($\omega_{\max}^{O},n_{\min}^{O}$) by enlarging the frequency $\omega_{z}$ linearly from $\omega_{\min}^{O}$ to $\omega_{\max}^{O}$. During this process, we ensure the mean value of $\omega_{z}A^{2}$, i.e., the mean phonon number and its entropy to be constant. Next, we apply the isochoric heating stroke from B ($\omega_{\max}^{O},n_{\min}^{O}$) to C ($\omega_{\max}^{O},n_{\max}^{O}$) by increasing the oscillation amplitude to $A_{\max}$ with $\omega_{z}=\omega_{\max}^{O}$. For the adiabatic expansion, we move from C ($\omega_{\max}^{O},n_{\max}^{O}$) to D ($\omega_{\min}^{O},n_{\max}^{O}$) by linearly decreasing $\omega_{z}$ from $\omega_{\max}$ to $\omega_{\min}$ with $\omega_{z}A^{2}$ staying unchanged. Finally, we perform the isochoric cooling from D ($\omega_{\min}^{O},n_{\max}^{O}$) to A ($\omega_{\min}^{O},n_{\min}^{O}$) by decreasing the oscillation amplitude to $A_{\min}^{O}$ with $\omega_{z}=\omega_{\min}^{O}$ remaining unchanged. To achieve a closed Otto cycle, we wait for the system to reach a steady state and return to its initial state.

**Fig. 2:**
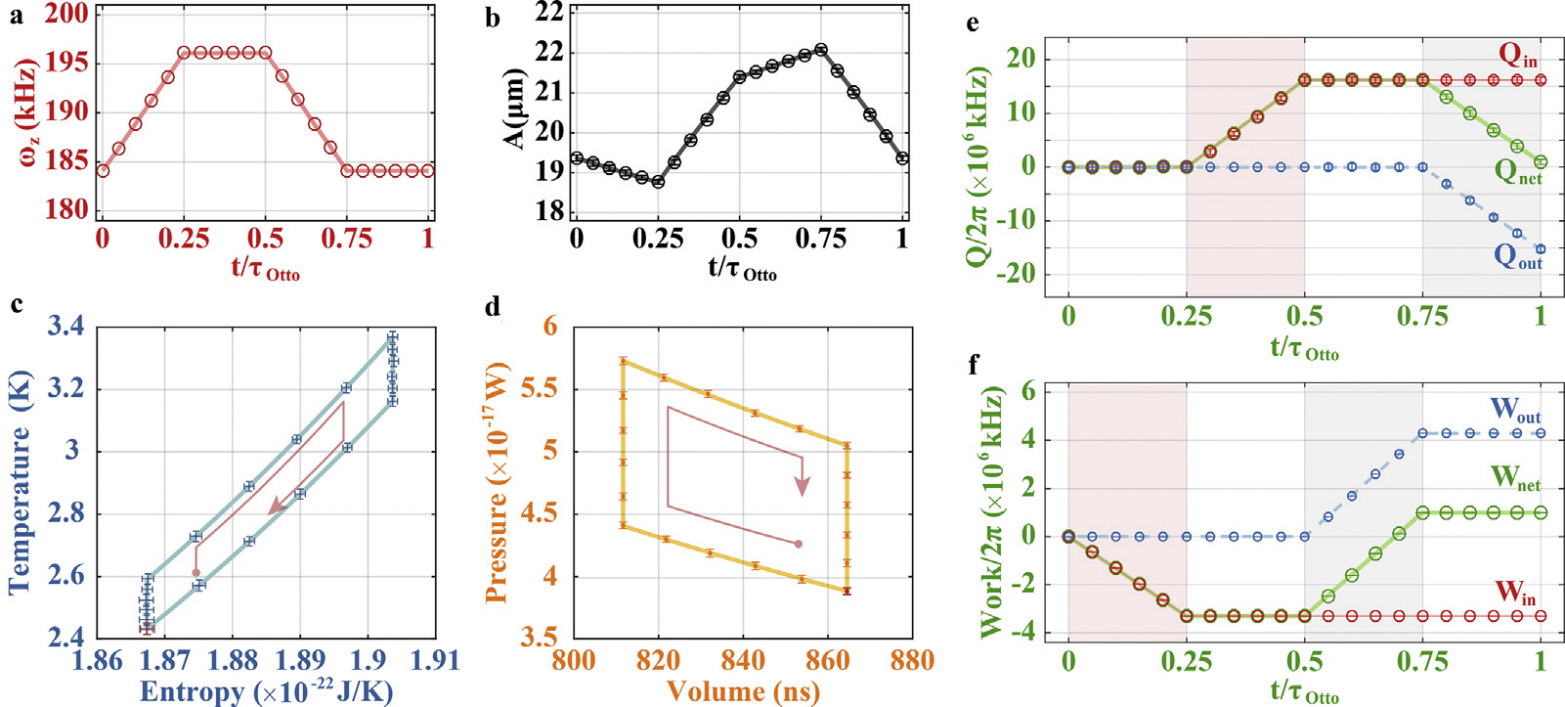
**Experimental results for the Otto cycle.** (a) Variation of the frequency $\omega_{z}$ of the phonon laser. (b) Variation of the amplitude A of the phonon laser. (c) Temperature-entropy diagram. (d) Pressure-volume diagram, where the enclosed area corresponds to the net work $W_{\mathrm{net}}$ during the Otto cycle. (e) Heat absorption $Q_{\mathrm{in}}$, heat release $Q_{\mathrm{out}}$, and net heat $Q_{\mathrm{net}}=Q_{\mathrm{in}}+Q_{\mathrm{out}}$, where the pink (gray) region represents the isochoric stroke for heating (cooling). These heats are calculated from the internal energy variations of the working substance. These calculations lead to $Q_{\mathrm{in}}>0$ and $Q_{\mathrm{out}}<0$. (f) Input work $W_{\mathrm{in}}$, output work $W_{\mathrm{out}}$, and net work $W_{\mathrm{net}}=W_{\mathrm{in}}+W_{\mathrm{out}}$, where the pink (gray) region represents the adiabatic stroke for compression (expansion). $\tau_{\mathrm{Otto}}$ means the total time of the four strokes. The solid lines and the dots with error bars correspond, respectively, to the theoretical simulation and experimental data, where the error bars are the statistical standard deviation of the experimental data from 40 measurements with each measurement time of 15 s.

Panels (a, b) of Fig. 2 present the engineered frequency $\omega_{z}$ and the measured amplitude A of the phonon laser throughout the Otto cycle. Based on these values, we plot the temperature-entropy (T-E) and pressure-volume (P-V) diagrams in panels (c) and (d), respectively, which indicate a standard procedure of the Otto cycle. The T-S diagram illustrates the isentropic strokes with constant entropy. The closed area in the P-V diagram represents the net work in an Otto cycle. Quantitatively, we acquire in Fig. 2e, f the net heat variation and the net work of the cycle. So the power of the Otto engine is $P_{O}/2\pi ≃9.925\times 10^{8}$ kHz/s, where $W_{\mathrm{net}}/2\pi =W_{O}/2\pi =9.925\times 10^{5}$ kHz and $\tau_{\mathrm{Otto}}=10$ ms. The corresponding Otto engine efficiency is given by $\eta_{O}=\left(W_{\mathrm{out}}+W_{\mathrm{in}}\right)/Q_{\mathrm{in}}=1-T_{D}/T_{\max}^{O}=1-T_{\min}^{O}/T_{B}≃6.12\%$, with $T_{\min}^{O}=T_{A}≃2.4317$ K, $T_{B}≃2.5902$ K, $T_{\max}^{O}=T_{C}≃3.3686$ K, and $T_{D}≃3.162$ K. In comparison with a previous heat engine experiment with efficiency η<0.045% [24], our experimentally observed Otto efficiency $\eta_{O}$ is much higher. The main reason for this difference is that, in our experiments, the tuning range of the frequency $\omega_{z}$ is much larger than that in Ref. [24]. Additionally, as we can experimentally control the frequency $\omega_{z}$ by the voltages of the SET electrodes, the maximum values of the SET electrode voltage determine the upper limit of the frequency $\omega_{z}^{\max}=2\pi \times 250$ kHz.

The equation calculating $\eta_{O}$ also suggests the possible improvement of the heat engine efficiency if one can set $T_{B}=T_{C}=T_{\max}^{O}$ and $T_{D}=T_{A}=T_{\min}^{O}$, implying that the isochoric strokes should be replaced by the isothermal strokes. It actually means the Carnot engine cycle. However, in this limit, no heat is drawn from the hot both or expelled to the cold bath, i.e., no work output from the engine.

The optical spins of an ion usually play an essential role in working substances in studying Otto cycles, e.g., the spins can output energy to its vibrational mode and detect the phonon number [19], [20]. Instead of these, here, we directly employ the vibrational mode as the working substances and measure the amplitude of the vibrational mode. It is due to the fact that the mean phonon number of phonon laser ($\approx 10^{6}$) is too large to measure. Such a large number of coherent phonons can avoid the influence of the thermal states and quantum fluctuations and ensure the exact Otto cycles in experiments. In addition, similar to Ref. [4], our experiment would possibly output the work by transferring the vibrational energy along the axial direction via the Coulomb interaction.

*Carnot cycles*. Now, we simulate Carnot cycles using the isothermal strokes. To carry out the isothermal strokes, which take the constant temperature $T=m\omega_{z}^{2}A^{2}/k_{B}$, we have to engineer A and $\omega_{z}$. Moreover, to explore the Carnot limit, which is relevant to the maximal and minimal temperatures of the baths, and to compare the efficiencies between the Carnot and Otto cycles, we set these two temperatures of the Carnot cycle to be the same as in the Otto cycle.

To accomplish the Carnot cycle as sketched in Fig. 1c, we prepare the vibrational mode of the ion to be initially in a steady state at E ($\omega_{\max}^{C},n_{\min}^{C}$), in which our working substance takes its maximal temperature $T=T_{\max}$. Then we accomplish the Carnot cycle with the following steps. We perform the isothermal expansion stroke from E ($\omega_{\max}^{C},n_{\min}^{C}$) to F ($\omega_{F},n_{\max}^{C}$) by decreasing $\omega_{z}$ linearly from $\omega_{\max}^{C}$ to $\omega_{F}$ along with a constant $\omega_{z}A=\sqrt{k_{B}T_{\max}/m}$. Then we execute the adiabatic expansion stroke from F ($\omega_{F},n_{\max}^{C}$) to G ($\omega_{\min}^{C},n_{\max}^{C}$) by linearly decreasing $\omega_{z}$ from $\omega_{F}$ to $\omega_{\min}^{C}$ along with an unchanged $\omega_{z}A^{2}$, followed by the isothermal compression from G ($\omega_{\min}^{C},n_{G}$) to H ($\omega_{H},n_{\min}^{C}$) by linearly increasing $\omega_{z}$ from $\omega_{\min}^{C}$ to $\omega_{H}$ with $\omega_{z}A=\sqrt{k_{B}T_{\min}/m}$ remaining unchanged. Finally, we implement the adiabatic compression stroke from H ($\omega_{H},n_{\min}^{C}$) to E ($\omega_{\max}^{C},n_{\min}^{C}$) by linearly increasing $\omega_{z}$ from $\omega_{H}$ to $\omega_{\max}^{C}$ with $\omega_{z}A^{2}$ being constant. The above Carnot cycle is closed since the end overlaps with the starting point of the isothermal stroke.

**Fig. 3:**
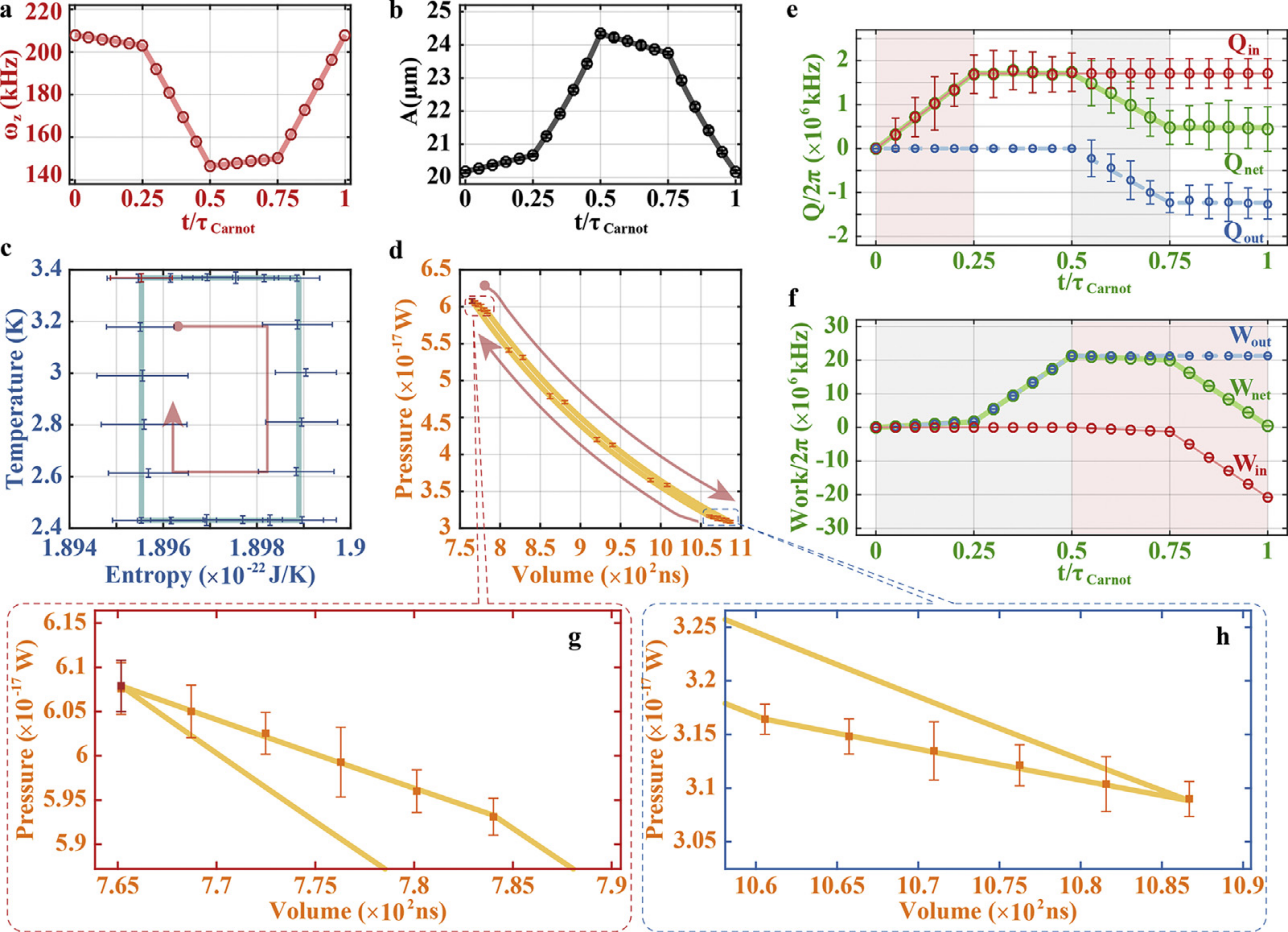
**Experimental results for the Carnot cycle.** (a) Variation of the frequency $\omega_{z}$ of the phonon laser. (b) Variation of the amplitude A of the phonon laser. (c) Temperature-entropy diagram. (d) Pressure-volume diagram, where the enclosed area corresponds to the net work $W_{\mathrm{net}}$. (e) Heat absorption $Q_{\mathrm{in}}$, heat release $Q_{\mathrm{out}}$, and net heat $Q_{\mathrm{net}}=Q_{\mathrm{in}}+Q_{\mathrm{out}}$, where the pink (gray) region represents the isothermal stroke for expansion (compression). These heat are calculated from the internal energy variations of the working substance. These calculations lead to $Q_{\mathrm{in}}>0$ and $Q_{\mathrm{out}}<0$. (f) Input work $W_{\mathrm{in}}$, output work $W_{\mathrm{out}}$, and net work $W_{\mathrm{net}}=W_{\mathrm{in}}+W_{\mathrm{out}}$. The gray (pink) region represents the work strokes, i.e., isothermal and adiabatic strokes, for expansion (compression). $\tau_{\mathrm{Carnot}}$ means the total time of the four strokes. Others are the same as in Fig. 2. g and h are zoom-in plots of the isothermal expansion and compression in d.

By varying $\omega_{z}$ and A as in panels (a, b) of Fig. 3, we acquire the T-E and P-V diagrams in panels (c) and (d), respectively. The T-E diagram demonstrates the isentropic strokes with constant values of entropy. The closed area in the P-V diagram represents the net work in the Carnot cycle. During this Carnot cycle, we can get the Carnot engine power $P_{C}/2\pi ≃4.759\times 10^{8}$ kHz/s, with $W_{\mathrm{net}}/2\pi =W_{C}/2\pi =4.759\times 10^{5}$ kHz and $\tau_{\mathrm{Carnot}}=10$ ms, and the Carnot engine efficiency $\eta_{C}=\left(W_{\mathrm{out}}+W_{\mathrm{in}}\right)/Q_{\mathrm{in}}=1-T_{\min}^{C}/T_{\max}^{C}≃27.85\%$, with $T_{\min}^{C}=T_{E}=T_{H}≃2.4308$ K, $T_{\max}^{C}=T_{F}=T_{G}≃3.3690$ K. This experimentally measured Carnot efficiency is nearly the value of the ideal result, which is mainly due to the fact that we have replaced the adiabatic strokes with the isentropic strokes in our implementation, ensuring equilibrium processes during the whole cycle.

A favorable feature of our Carnot cycle is that we can suppress the thermal and quantum fluctuations of the heat cycles [1], [2], [4], [6], [32] by the phonon laser effect [33], [34], [35], [36], [37], [38], [39], [40]. For this reason, we can simulate the exact Carnot cycles and reach the Carnot efficiency, which has never been achieved in previous experiments [6], [53].

The two sets of experiments presented above demonstrate clearly that the Carnot efficiency is larger than the Otto one when the two cycles share the same high and low temperature limits of the baths (i.e., $T_{\min}^{O}≃T_{\min}^{C}$, $T_{\max}^{O}≃T_{\max}^{C}$), which is consistent with the result of conventional thermodynamics. To ensure the same temperature limits for the two cycles, in our experiment, we have to set the frequency variation in the Carnot cycle to be much larger than that in the Otto cycle. Moreover, the tunable range of the trap frequency, from 100 kHz to 300 kHz, ensures that the amplitude variation δA is identifiable from the CCD image. This helps our direct observation of thermodynamic processes in the experiment, although we have exactly evaluated δA by fitting the scattered photons recorded by the PMT.

## 4 Conclusion

In conclusion, we have experimentally simulated the classical heat engine cycles in a trapped-ion system by coherently manipulating the vibrational mode. To our knowledge, this is the first experimental demonstration of the standard processes of classical Carnot and Otto engine cycles at the single-atom level. Following this idea, our further works would focus on the work extractions from these heat engines and also simulation of other classical heat engines, such as the Stirling cycles [54] and the Diesel cycles [55], which are less famous while with wide application. Our simulation might give deeper insight into the commonalities and advantages of different heat cycles in thermodynamic effects.

Since the single trapped-ion system has already been employed to study quantum thermodynamics, using the spin degrees of freedom and/or the vibrational modes [11], [18], [19], [56], [57], [58], [59], [60], [61], our work here indicates the interesting possibility of using the single-ion platform to explore both classical and quantum thermodynamics. Moreover, it is possible to extend our method and results to other vibrational systems, such as the cantilevers, which would open new possibilities towards developing phonon lasers for practical application via optical manipulation techniques.

## Declaration of competing interest

The authors declare that they have no conflicts of interest in this work.

## Appendix A Supplementary materials

Supplementary Data S1 Supplementary Raw Research Data. This is open data under the CC BY license http://creativecommons.org/licenses/by/4.0/ Supplementary Data S1
