# Supplementary material for: Exact simulation of classical heat engine cycles using single-ion phonon laser
Source: Fundam Res. 2024 Mar 5;6(1):149–54. doi: 10.1016/j.fmre.2024.01.008 (PMC12869772; doi:10.1016/j.fmre.2024.01.008)
Supplement: Supplementary Data S1 — Supplementary Raw Research Data. This is open data under the CC BY license http://creativecommons.org/licenses/by/4.0/ [file mmc1.pdf]

SUPPLEMENT

I. HAMILTONIAN OF THE WORKING SUBSTANCE

As the working substance is the vibrational mode of the single ion, to study its thermodynamics, we present in this section the derivation of the Hamiltonian of the vibrational mode.

To study the dynamics of the single ion, we employed the Langevin equations in the main text as:

$$m\frac{d^2}{dt^2}z + m\gamma_{\text{eff}}\frac{d}{dt}z + m\omega_{\text{z}}^2 z = F\sin(\omega_l t) \tag{2}$$

which can be rewritten as:

$$\begin{aligned} \frac{d}{dt}p &= -m\omega_{\text{z}}^2 z - \gamma_{\text{m}} p + F\sin(\omega_{\text{l}} t) \\ \frac{d}{dt}z &= \frac{p}{m} \end{aligned} \tag{3}$$

where m is the mass of the ion, $\gamma_{\text{eff}} > 0(\gamma_{\text{eff}} < 0)$ is the effective loss (gain) rate related to the driving optical fields, ω_{z} is the z-axis frequency of the trap, F and ω_l are, respectively, the strength and frequency of the injection-locking signal, z and $p = m\frac{dz}{dt}$ are the position and momentum of the ion, respectively. After setting $\omega_l = \omega_{\text{z}}$, we can write the effective Hamiltonian of the oscillator as:

$$H = m\omega_{\text{z}}^2 A^2 = \langle n \rangle \hbar\omega_{\text{z}} \tag{4}$$

where the amplitude A of the oscillator and the mean phonon number $\langle n \rangle$ are key quantities to be measured in our experiment.

II. DEFINITIONS OF THERMODYNAMIC QUANTITIES

In this section, we define the thermodynamic quantities following Refs. [8, 59]. For example, we define the mean phonon number and temperature in subsection II.1, and then we present the definitions for heat and work in subsection II.2. Besides, we give the partition function and entropy and show how to understand the definition of volume and pressure.

II.1 Mean phonon number and temperature

From Eq. 4, we define the mean phonon number:

$$\langle n \rangle = m\omega_z A^2/\hbar$$

According to Ref.[28], the temperature of the phonon laser can be defined as:

$$T_{\text{eff}} = \frac{\langle n \rangle \hbar\omega_z}{k_B log(1 + n_{th}^{-1})n_{th}}$$

with thermal phonon number n_{th} and the Boltzmann constant k_B.

In the case of $n_{th} \gg 1$, we can get $log(1 + n_{th}^{-1})n_{th} \approx 1$. Then the effective temperature T_{eff} of the working substance can be written as:

$$k_B T_{\text{eff}} = m\omega_z^2 A^2 = \langle n \rangle \hbar\omega_z$$

Thus we can get the effective temperature of the vibrational mode as:

$$T_{\text{eff}} = m\omega_z^2 A^2/k_B = \langle n \rangle \hbar\omega_z/k_B \tag{5}$$

which is similar to Ref. [28], because our working substance is in a thermal coherent state of the photon laser. The effective temperature T_{eff} (5) represents the temperature of an equivalent thermal state [28] by assuming that the coherent phonon number dominates the system ($\tilde{n}_{ph} \gg n_{th}$) and the thermal phonon number is large enough ($n_{th} \gg 1$).

II.2 Heat and work

According to the Hamiltonian (4), we write the energy of the vibrational mode as:

$$U = m\omega_z^2 A^2 = \langle n \rangle \hbar\omega_z \tag{6}$$

which means:

$$\begin{aligned} dU &= 2m\omega_z^2 A dA + 2mA^2\omega_z d\omega_z \\ &= \hbar\omega_z d\langle n \rangle + \langle n \rangle \hbar d\omega_z \end{aligned}$$

In classical thermodynamics, the first law of thermodynamics is $dU = dQ + dW$, with the heat dQ and the work dW. Due to the heat $dQ = TdS$ depending on the entropy $S = -k_B \sum_n P_n \ln P_n$, we identify:

$$\begin{aligned} dQ &= \hbar\omega_z d\langle n \rangle = 2m\omega_z^2 A dA \\ dW &= \langle n \rangle \hbar d\omega_z = 2mA^2\omega_z d\omega_z \end{aligned} \tag{7}$$

According to Eq. 7, the increase (decrease) of the amplitude A represents the absorbed (released) heat Q_{in} (Q_{out}) and the increase (decrease) of the frequency ω_{z} implies the input (output) work. Moreover, in the closed cycles, our numerical simulations show that heat $|dQ|$ is equivalent to work $|dW|$ (see main text Figs. 2 and 3).

II.3 Partition function and entropy

The partition function of the working substance is given by [59]:

$$\begin{aligned} Z_{\text{eff}} &= \frac{1}{h}\int\int \exp[-\frac{H}{k_B T_{\text{eff}}}]dxdp = \frac{1}{h}\int\int dxdp\exp[-\frac{\frac{1}{2}m\omega_{\text{z}}^2x^2+\frac{p^2}{2m}}{k_B T_{\text{eff}}}] \\ &= \frac{1}{4h}\sqrt{\frac{2k_B T_{\text{eff}}\pi}{m\omega_{\text{z}}^2}}\sqrt{2k_B T_{\text{eff}} m\pi} = \frac{k_B T_{\text{eff}}}{2\hbar\omega_{\text{z}}} \end{aligned}$$

and the entropy is written as:

$$\begin{aligned} S_0 &= k_B[\ln Z_{eff} - \frac{1}{k_B T_{\text{eff}}}\frac{\partial \ln Z_{\text{eff}}}{\partial(\frac{1}{k_B T_{\text{eff}}})}] \\ &= k_B(\ln Z_{\text{eff}} + 1) \\ &= k_B(\ln\frac{k_B T_{\text{eff}}}{2\hbar\omega_{\text{z}}} + 1) = S - k_B\ln 2 \end{aligned}$$

For convenience, we employ:

$$S = k_B(1+\ln\frac{k_B T_{\text{eff}}}{\hbar\omega_{\text{z}}}) \tag{8}$$

to calculate the entropy in the main text, following the definition in [8].

II.4 Volume and pressure

The harmonic volume of the vibrational mode is defined as:

$$V = AL = A\frac{m\lambda}{2} = A\frac{mv}{4\pi}\frac{1}{\omega_{\text{z}}} \sim \frac{1}{\omega_{\text{z}}}$$

where A is the constant area of the vibrational mode, L is the tunable length, λ is the wavelength of the vibrational mode, and v is the group velocity of the vibrational mode. We set $V = \frac{1}{\omega_{\text{z}}}$ in the main text.

The pressure follows:

$$P = -\left(\frac{\partial U}{\partial V}\right)_S = -\frac{\partial(m\omega_{\text{z}}^2A^2)}{\partial\omega_{\text{z}}^{-1}} = 2m\omega_{\text{z}}^3A^2 \sim m\omega_{\text{z}}^3A^2$$

For simplicity, we set $P = m\omega_{\text{z}}^3A^2$.

III. COMPARISON OF ADIABATIC STROKES WITH ISENTROPIC STROKES

In our experiment for heat cycles, since the working substance (i.e., the ion's vibrational state) cannot be fully separated from its environment, experimentally implementing the adiabatic strokes is hard. Hence, we replace the adiabatic strokes with the isentropic strokes, which are physically identical but technically available. We present below a comparison of these two strokes.

From Eqs. 5 and 8, we can obtain the mean phonon number [28]:

$$\langle n \rangle = k_B T_{\mathrm{eff}}/\hbar\omega_{\mathrm{z}} = m\omega_{\mathrm{z}}A^2/\hbar$$

and the variation of the entropy between the mean phonon number $\langle n \rangle_{\mathrm{i}}$ for the initial state and $\langle n \rangle_{\mathrm{f}}$ for the final state as [8]:

$$\Delta S = k_B \ln \langle n \rangle_{\mathrm{f}} - k_B \ln \langle n \rangle_{\mathrm{i}} = k_B \ln \frac{\langle n \rangle_{\mathrm{f}}}{\langle n \rangle_{\mathrm{i}}} \tag{9}$$

The adiabatic strokes require the mean phonon number to keep constant, i.e., $\langle n \rangle_{\mathrm{i}} = \langle n \rangle_{\mathrm{f}}$. Then we rewrite the above equation as:

$$\Delta S = k_B \ln \frac{\langle n \rangle_{\mathrm{f}}}{\langle n \rangle_{\mathrm{i}}} = 0 \tag{10}$$

It implies that we can physically replace the adiabatic strokes with the isentropic strokes. Experimentally, this is accomplished by keeping $\omega_{\mathrm{z}}A^2$ constant by appropriately adjusting the frequency and strength of the blue-detuned laser beam.

III. THE OTTO CYCLE

In this section, we evaluate the efficiency of the Otto cycle and its maximum value.

1. Efficiency of the Otto cycle

In the idea of adiabatic processes, as plotted in the upper panel of the main text Fig. 1c, the mean phonon number remains constant, i.e., $\langle n_A \rangle = \langle n_B \rangle$, and $\langle n_C \rangle = \langle n_D \rangle$.

In classical thermodynamics, there is an automatic energy transfer from the high-temperature system to the low-temperature one, i.e., the unidirectional energy flow. Thus,

we define the energy absorption from the hot bath in the isochoric heating stroke as:

$$Q_{\text{in}} = (\langle n_C \rangle - \langle n_B \rangle)\hbar\omega_{\max} > 0$$

During the isochoric cooling stoke, the energy is released from the system to the cold bath:

$$Q_{\text{out}} = (\langle n_A \rangle - \langle n_D \rangle)\hbar\omega_{\min} = -(\langle n_C \rangle - \langle n_B \rangle)\hbar\omega_{\min} < 0$$

where $\omega_{\max}$ and $\omega_{\min}$ are the maximum and minimum effective frequencies in the isochoric heating and cooling strokes, respectively. So we obtain the net work W_{O} and efficiency η_{O} of the Otto engine as:

$$W_{\text{O}} = Q_{\text{out}} + Q_{\text{in}} \tag{11}$$

and

$$\eta_{\text{O}} = \frac{W_{\text{O}}}{Q_{\text{in}}} = 1 + \frac{Q_{\text{out}}}{Q_{\text{in}}} = 1 - \frac{\omega_{\min}}{\omega_{\max}} \tag{12}$$

During the adiabatic processes, the mean phonon number and the thermal state are constant, while the effective frequency is shifted from $\omega_{\min}$ ($\omega_{\max}$) to $\omega_{\max}$ ($\omega_{\min}$). It reminds us of the possibility of writing the Otto engine efficiency with temperatures of different adiabatic processes by Eq. 5.

In the adiabatic compression stroke, we can write this efficiency with the temperatures at points A and B as:

$$\eta_{\text{O}}^{AB} = 1 - \frac{\omega_{\min}}{\omega_{\max}} = 1 - \frac{\langle n_A \rangle \hbar\omega_{\min}/k_B}{\langle n_B \rangle \hbar\omega_{\max}/k_B} = 1 - \frac{T_A}{T_B} \tag{13}$$

and the efficiency in the adiabatic expansion stroke with the temperatures at points C and D is:

$$\eta_{\text{O}}^{CD} = 1 - \frac{\omega_{\min}}{\omega_{\max}} = 1 - \frac{\langle n_D \rangle \hbar\omega_{\min}/k_B}{\langle n_C \rangle \hbar\omega_{\max}/k_B} = 1 - \frac{T_D}{T_C} \tag{14}$$

In a closed Otto cycle, the efficiency is $\eta_{\text{O}}^{AB} = \eta_{\text{O}}^{CD}$. Considering the lowest temperature $T_A = T_{\min}$ and the highest one $T_C = T_{\max}$, we can rewrite the above efficiencies as:

$$\eta_{\text{O}} = \eta_{\text{O}}^{AB} = \eta_{\text{O}}^{CD} = 1 - \frac{T_D}{T_{\max}} = 1 - \frac{T_{\min}}{T_B} \tag{15}$$

2. Parameters for the maximum efficiency of the Otto cycle

Here we consider the maximum efficiency, which takes the maximum work [60]. From Eq. 15, we have:

$$\begin{aligned} \eta_{\mathrm{O}}^{AB} &= \eta_{\mathrm{O}}^{CD} \\ 1-\frac{T_D}{T_{\max}} &= 1-\frac{T_{\min}}{T_B} \\ \frac{T_D}{T_{\max}} &= \frac{T_{\min}}{T_B} \\ T_{\max}T_{\min} &= T_B T_D \leq \tfrac{T_B^2+T_D^2}{2} \end{aligned} \tag{16}$$

The above inequality in Eq. 16 reduces to the equality when $T_B = T_D = \sqrt{T_{\max}T_{\min}}$. In our case, the efficiency η_{O} reaches its maximum value as:

$$\eta_{\mathrm{O}}^{\max} = 1-\frac{\omega_{\min}}{\omega_{\max}} = 1-\sqrt{\frac{T_{\min}}{T_{\max}}} \tag{17}$$

IV. THE CARNOT CYCLE

In this section, we present the efficiency and the maximum net work of the Carnot cycle and derive the Curzon-Ahlborn efficiency.

1. Efficiency of the Carnot cycle

During the isothermal processes, as in the lower panel of the main text Fig. 1c, the temperature remains constant, i.e., $\langle T_E\rangle = \langle T_F\rangle = T_{\max}$, and $\langle T_G\rangle = \langle T_H\rangle = T_{\min}$. During the adiabatic processes, the entropy remains constant, i.e., $S_F = S_G$ and $S_H = S_E$.

In classical thermodynamics, the net work done in the Carnot cycle is defined as:

$$\begin{aligned} W_{\mathrm{C}} &= Q_{\mathrm{in}} + Q_{\mathrm{out}} \\ &= T_{\max}(S_F - S_E) + T_{\min}(S_H - S_G) \end{aligned}$$

For example, in the isothermal expansion from E to F, we acquire the net work as:

$$\begin{aligned} W_{\mathrm{C}}^{EF} &= T_{\max}(S_F - S_E) + T_{\min}(S_E - S_F) \\ &= (T_{\max} - T_{\min})(S_F - S_E) \\ &= k_B(T_{\max} - T_{\min}) \ln \frac{T_{\max}}{T_{\min}}\frac{\omega_E}{\omega_F} \end{aligned}$$

Correspondingly, the net work in the isothermal compression from G to H is:

$$\begin{aligned} W_{\mathrm{C}}^{GH} &= T_{\max}(S_G - S_H) - T_{\min}(S_G - S_H) \\ &= k_B(T_{\max} - T_{\min}) \ln \frac{T_{\max}}{T_{\min}} \frac{\omega_H}{\omega_G} \end{aligned}$$

For a closed Carnot cycle, we have $W_{\mathrm{C}} = W_{\mathrm{C}}^{GH} = W_{\mathrm{C}}^{EF}$.

In the Carnot cycle, the absorbed heat is:

$$Q_{in} = T_{\max}(S_F - S_E)$$

Then we can obtain the efficiency of the Carnot engine as:

$$\eta_{\mathrm{C}} = \frac{W_{\mathrm{C}}}{Q_{\mathrm{in}}} = \frac{(T_{\max} - T_{\min})(S_F - S_E)}{T_{\max}(S_F - S_E)} = 1 - \frac{T_{\min}}{T_{\max}} \tag{18}$$

2. Parameters for maximum net work of the Carnot cycle

Considering the lowest frequency $\omega_G = \omega_{\min}$ and the highest one $\omega_E = \omega_{\max}$, we obtain:

$$\begin{aligned} (T_{\max} - T_{\min}) \ln \frac{T_{\max}}{T_{\min}} \frac{\omega_H}{\omega_G} &= (T_{\max} - T_{\min}) \ln \frac{T_{\max}}{T_{\min}} \frac{\omega_E}{\omega_F} \\ \frac{T_{\max}}{T_{\min}} \frac{\omega_H}{\omega_G} &= \frac{T_{\max}}{T_{\min}} \frac{\omega_E}{\omega_F} \\ \frac{\omega_H}{\omega_G} = \frac{\omega_H}{\omega_{\min}} &= \frac{\omega_{\max}}{\omega_F} = \frac{\omega_E}{\omega_F} \\ \omega_{\max}\omega_{\min} &= \omega_H \omega_F \leq \frac{\omega_H^2 + \omega_F^2}{2} \end{aligned}$$

The above inequality in Eq. 16 turns to be an equality if and only if $\omega_H = \omega_F = \sqrt{\omega_{\max}\omega_{\min}}$. In this situation, the net work of the Carnot cycle reaches its maximum value.

3. The Curzon-Ahlborn efficiency of the Carnot cycle

In a closed Carnot cycle, the total entropy of the environment must increase, so we have:

$$\oint dS = -\frac{Q_{\mathrm{in}}}{T_{\max}} - \frac{Q_{\mathrm{out}}}{T_{\min}} \geqslant 0$$

and

$$Q_{\mathrm{out}} \leqslant -Q_{\mathrm{in}} \frac{T_{\min}}{T_{\max}} \tag{19}$$

Inserting Eq. 19 into Eq. 18, we acquire:

$$\eta_{\mathrm{C}}^{\mathrm{r}} = \frac{W_{\mathrm{C}}}{Q_{\mathrm{in}}} = \frac{Q_{\mathrm{in}} + Q_{\mathrm{out}}}{Q_{\mathrm{in}}} \leqslant \frac{Q_{\mathrm{in}} - Q_{\mathrm{in}} \frac{T_{\min}}{T_{\max}}}{Q_{\mathrm{in}}} = 1 - \frac{T_{\min}}{T_{\max}}$$

To achieve Curzon-Ahlborn efficiency, we assume the working substance of the heat engine interacts with the heat bath via a thermal conductor. The thermal conductor takes a heat capacity $C_{\rm T}$ and provides a lower temperature $T_{\rm h}$ ($T_{\rm h} < T_{\rm max}$). Then we can write the heat absorption from the heat bath as:

$$Q^{\rm r}_{\rm in} = C_{\rm T}(T_{\rm max} - T_{\rm h})$$

and the corresponding work as:

$$W^{\rm r}_{\rm C} = \eta_{\rm C} Q_{\rm in} = (1 - \frac{T_{\rm min}}{T_{\rm h}}) C_{\rm T}(T_{\rm max} - T_{\rm h})$$

Thus we have:

$$\begin{aligned} \frac{dW^{\rm r}_{\rm C}}{dT_{\rm h}} &= C_{\rm T} \frac{d}{dT_{\rm h}}(T_{\rm max} + T_{\rm min} - T_{\rm h} - \frac{T_{\rm max}T_{\rm min}}{T_{\rm h}}) \\ &= -C_{\rm T}(1 - T_{\rm max}T_{\rm min}T_{\rm h}^{-2}) \end{aligned}$$

292 and the optimal temperature for $\frac{dW^{\rm r}_{\rm C}}{dT_{\rm h}} = 0$ is $T_{\rm h} = \sqrt{T_{\rm max}T_{\rm min}}$.

Finally, we obtain the Curzon-Ahlborn efficiency of the Carnot cycle as:

$$\eta_{\rm CA} = 1 - \frac{T_{\rm min}}{T_{\rm h}} = 1 - \frac{T_{\rm min}}{\sqrt{T_{\rm max}T_{\rm min}}} = 1 - \sqrt{\frac{T_{\rm min}}{T_{\rm max}}}$$

293 ## V. PHYSICAL PICTURES FOR HEAT EXCHANGE

294 To further understand the heat exchange processes, we show below some physical pictures
295 of these processes.

Although it is possible to experimentally simulate the heat engine cycles with the thermal states of the ion, the fluctuations can seriously influence the efficiencies and lead to the efficiencies deviating from the ideal ones, as reported previously [1, 2, 4, 6, 32]. To acquire accurate experimental efficiencies, we try to reduce the influence of quantum fluctuations by increasing the coherent phonon number $\tilde{n}_{ph}$ for:

$$\langle n \rangle = \tilde{n}_{ph} + n_{th} = \tilde{n}_{ph}(1 + \frac{n_{th}}{\tilde{n}_{ph}}) \simeq \tilde{n}_{ph} \qquad (20)$$

296 Therefore, both the absorbed heat $Q_{\rm in}$ and released heat $Q_{\rm out}$ correspond to the increase and
297 decrease of the mean phonon number $\langle n \rangle \propto \omega_{\rm z} A^2$ (see main text Fig. 1c), respectively. In
298 other words, we can observe a similar energy exchange as the phonon laser via the variations
299 of the amplitude A (see main text Figs. 2 and 3) as described in Eq. 7.

VI. OTHER RELATIONS BETWEEN THE PARAMETERS

Now we show how to control the amplitude A by the intensity ratio between the blue-detuned and red-detuned beams and present how to operate the trap frequency by electrode voltages.

Define $z = Ae^{i\omega_{\rm l}t} + Be^{-i\omega_{\rm l}t}$, we obtain:

$$\begin{aligned} \frac{d}{dt}z &= Ai\omega_{\rm l}e^{i\omega_{\rm l}t} - Bi\omega_{\rm l}e^{-i\omega_{\rm l}t} \\ \frac{d^2}{dt^2}z &= -A\omega_{\rm l}^2e^{i\omega_{\rm l}t} - B\omega_{\rm l}^2e^{-i\omega_{\rm l}t} \end{aligned} \tag{21}$$

and thus Eq. (S1) can be rewritten as:

$$m(-A\omega_{\rm l}^2e^{i\omega_{\rm l}t} - B\omega_{\rm l}^2e^{-i\omega_{\rm l}t}) + im\gamma_{\rm eff}(A\omega_{\rm l}e^{i\omega_{\rm l}t} - B\omega_{\rm l}e^{-i\omega_{\rm l}t}) + m\omega_{\rm z}^2(Ae^{i\omega_{\rm l}t} + Be^{-i\omega_{\rm l}t}) = -i\frac{F}{2}(e^{i\omega_{\rm l}t} - e^{-i\omega_{\rm l}t}) \tag{22}$$

Then, we resort the above equations with different frequencies ($e^{i\omega_{\rm l}t}$ and $e^{i\omega_{\rm l}t}$) as:

$$\begin{aligned} -Am\omega_{\rm l}^2 + iA\omega_{\rm l}m\gamma_{\rm eff} + m\omega_{\rm z}^2A &= -i\frac{F}{2} \\ -Bm\omega_{\rm l}^2 - iBm\omega_{\rm l}\gamma_{\rm eff} + m\omega_{\rm z}^2B &= i\frac{F}{2} \end{aligned}$$

As a result, the corresponding solutions are:

$$z = \frac{-iF/2m}{(\omega_{\rm z}^2 - \omega_{\rm l}^2) + i\omega_{\rm l}\gamma_{\rm eff}}e^{i\omega_{\rm l}t} + \frac{iF/2m}{(\omega_{\rm z}^2 - \omega_{\rm l}^2) - i\omega_{\rm l}\gamma_{\rm eff}}e^{-i\omega_{\rm l}t}$$

and

$$A = \frac{F/2m}{\sqrt{(\omega_{\rm z}^2 - \omega_{\rm l}^2)^2 + \omega_{\rm l}^2\gamma_{\rm eff}^2}}$$

In the case of $\omega_{\rm l} = \omega_{\rm z}$, the above amplitude can be reduced to:

$$A = \frac{F}{2m\omega_{\rm z}\gamma_{\rm eff}}$$

The experimental observation in Fig. 4 shows that the effective decay rate $\gamma_{\rm eff}$ is a linear function of the ratio $\ln\frac{I_b}{I_r}$, where I_b and I_r are, respectively, the strengths of the blue- and red-detuned laser fields. By fitting the experimental results, we take $\gamma_{\rm eff} = k_\gamma/(\ln\frac{S_1}{S_2} + d)$ with the constants k_γ and d. As a result, we can rewrite the amplitude as:

$$A = \frac{F}{2m\omega_{\rm z}k_\gamma}(\ln\frac{I_b}{I_r} + d)$$

Next, we present the details of the experimental control of the electrode voltage, including a sketch of the surface-electrode trap in Fig. 5 and the frequency of the phonon laser with respect to the voltages of the electrodes in Table. .

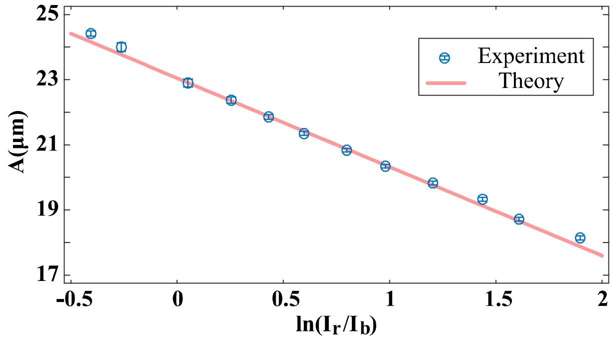

FIG. 4. **The linear relation between A and $\ln \frac{I_b}{I_r}$.** The parameters are set as $\omega_z = 2\pi \times 183.37$ kHz, $\frac{F}{2m\omega_1 k_\gamma} = -2.725$ μm and $d = -8.4584$.

TABLE I. Voltages and trap frequency

No.	Voltages (V) in electrodes #3 and #8	Voltages (V) in electrodes #5 and #6	$\omega_z/2\pi$ (kHz)
1.	-2.338	-0.178	224.3
2.	-1.738	-0.041	214.9
3.	-1.138	0.091	204.8
4.	-0.538	0.266	194.5
5.	0.062	0.364	183.5
6.	0.622	0.49	172.4
7.	1.142	0.605	161.9
8.	1.642	0.721	150.6
9.	1.978	0.799	142.8

VII ACQUIREMENT OF A FROM THE PMT-RECORDED PHOTONS

Now, we show how to get the amplitude A from the PMT-recorded photons.

We introduce the approach to acquire the value of A by fitting the recorded photons scattered from the ion [45]. We assume that the amplitude A and phase φ of the ion oscillation are constants during our measurement. Under the irradiation of the 397-nm laser beams, the scattering rate ρ_j at time t is given by [61]

$$\rho_j(t) = \frac{\Gamma s_j/(4\pi)}{1 + s_j + 4\left[\frac{\Delta_j - k_j\omega_i A\cos(\omega_i t + \varphi)}{\Gamma}\right]^2} \tag{23}$$

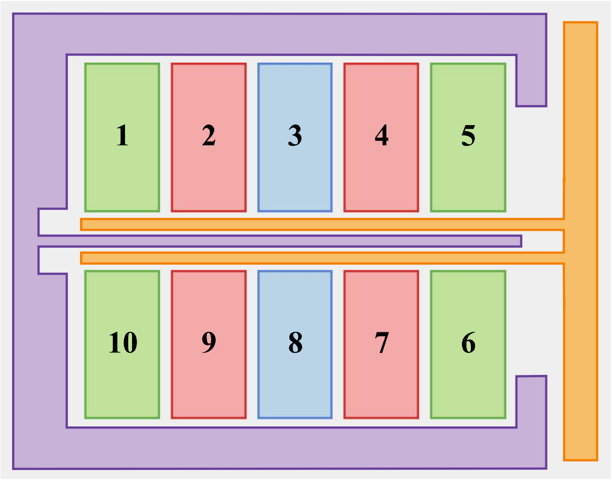

FIG. 5. **Sketch of the surface-electrode trap**. The electrodes are labeled by numbers for convenience of description.

where Γ is the decay rate of $P_{1/2}$, k_j is the wave vector, Δ_j means the detuning and s_j represents the saturation parameter. Due to the two 397-nm laser beams with different detunings in our experiment, the total scattering rate is $\rho(t) = \rho_1(t) + \rho_2(t)$. Since the experimental data include the influence of different noises in the experimental system, we employ the Gaussian term $G(t)$ to fit the curve of $P(t)$ by a convolution function:

$$P(t) = \alpha\rho(t) * G(t) + \beta \tag{24}$$

where the Gaussian term $G(t) = (1/\sqrt{2\pi}\sigma_t)exp[-t^2/(2\sigma_t^2)]$ takes the degree of time dispersion σ_t. α and β represent the factors regarding the measurement time, the fluorescence collection efficiency, the background photons and the number of unit time intervals as well as the signal-to-noise ratio relevant to the background light. These parameters can be determined experimentally before acquiring the oscillation amplitude A [45].

TABLE II. Parameter variation in the Otto cycle

Steps	Strokes	Constant items	ω_z	Amplitude A
A→B	Adiabatic compression stroke	$\langle n \rangle \propto \omega_z A^2$	$\nearrow$	$\searrow$
B→C	Isochoric heating stroke	ω_z	$\backslash$	$\nearrow$
C→ D	Adiabatic expansion stroke	$\langle n \rangle \propto \omega_z A^2$	$\searrow$	$\nearrow$
D→A	Isochoric cooling stroke	ω_z	$\backslash$	$\searrow$

TABLE III. Parameter variation in the Carnot cycle

Steps	Strokes	Constant items	ω_z	Amplitude A
E→F	Isothermal expansion stroke	$T \propto \omega_z^2 A^2$	$\searrow$	$\nearrow$
F→ G	Adiabatic expansion stroke	$\langle n \rangle \propto \omega_z A^2$	$\searrow$	$\nearrow$
G→ H	Isothermal compression stroke	$T \propto \omega_z^2 A^2$	$\nearrow$	$\searrow$
H→ E	Adiabatic compression stroke	$\langle n \rangle \propto \omega_z A^2$	$\nearrow$	$\searrow$

VIII. TABLES FOR PARAMETER VARIATIONS IN OTTO AND CARNOT CYCLES

IX. EFFICIENCIES OF HEAT ENGINE CYCLES VERSUS THE EXPERIMENTAL PARAMETERS

Here we would like to discuss the externally controllable parameters of our experimental platform and present the corresponding efficiencies of the heat cycles versus the heat baths and trap frequencies.

According to the discussions and the Tables above, in the experimental limits, we can modulate the trap frequency $\omega_z/2\pi$ from 100 kHz to 250 kHz and control the amplitude A between 15 μm to 40 μm. In the present experiment, the trap frequency $\omega_z/2\pi$ ranges from 142.8 kHz to 224.3 kHz, and the amplitude A ranges from 18 μm to 24 μm. Thus we have the corresponding temperature range from 2.43 K to 3.37 K. We have simulated the efficiencies of the Otto and Carnot cycles in Fig. 6.

In the Otto cycles, when the minimum trap frequency $\omega_z^{\min}$ is fixed, the efficiency is only dependent on the maximum trap frequency $\omega_z^{\max}$ [see Eq. 12]. On the other hand, the

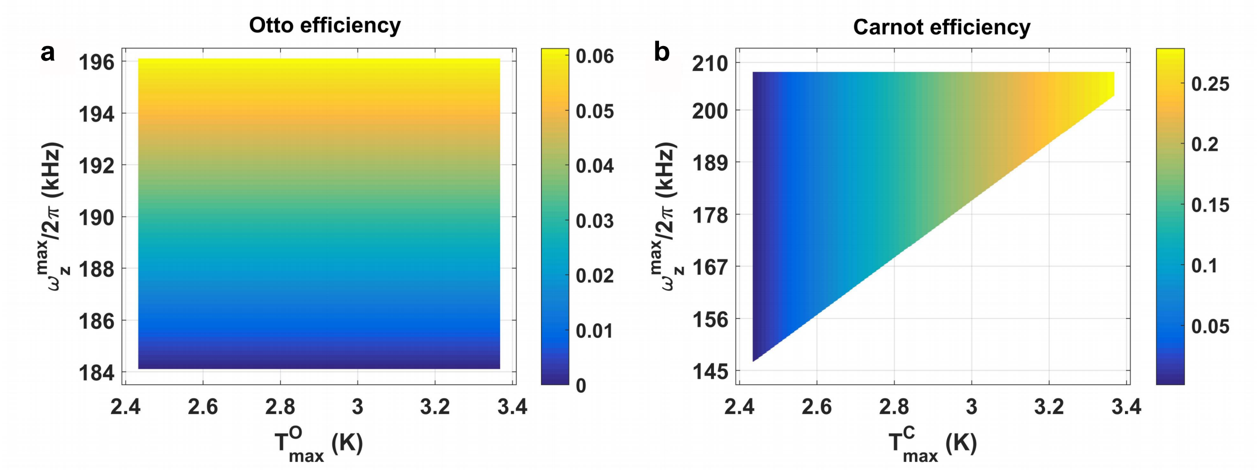

FIG. 6. **Theoretical efficiency of the Otto and Carnot cycles.** (a) Efficiency of the Otto cycles versus the temperature of the hot bath $T^{\rm O}_{\rm max}$ and the trap frequency $\omega_{\rm z}^{\rm max}$. (b) Efficiency of the Carnot cycles versus the temperature of the hot bath $T^{\rm C}_{\rm max}$ and the maximum trap frequency $\omega_{\rm z}^{\rm max}$. Here we set the cold bath takes the temperature $T^{\rm O}_{\rm min} = T^{\rm C}_{\rm min}$ =2.43 K, and the minimum trap frequency $\omega_{\rm z}^{\rm min} = 2\pi \times 142.8$ kHz.

efficiency of the closed Otto cycle is determined by the temperatures of the starting and ending points of the adiabatic processes, rather than those of the hot and cold baths [see Eqs. 13 and 14]. Thus, this efficiency is independent from the maximum temperature $T^{\rm O}_{\rm max}$ of the Otto cycles. As a result, we can find that the Otto efficiency works as a function of the maximum trap frequency.

In the Carnot cycles, under the action of the constant temperature of the cold bath, the efficiency varies with the temperature of the hot bath $T^{\rm C}_{\rm max}$ as in Eq. 18. The physical picture is the following. The Carnot cycles contain isothermal processes and adiabatic processes. Due to no entropy variation in the adiabatic processes, the increase and decrease of the entropy must be equal in the closed Carnot cycle. Therefore, the efficiency of the Carnot cycle only depends on the temperatures of the baths. Moreover, due to the experimental limit of the amplitude A, by increasing the temperature $T^{\rm C}_{\rm max}$, the maximum values of the amplitude A would go beyond the upper limit of amplitude ($> 40\ \mu m$), and the corresponding efficiencies are ignored for this reason.
